# Influencing nursing students’ perceptions of community care with curriculum-redesign; a quasi-experimental cohort study

**DOI:** 10.1186/s12909-019-1733-5

**Published:** 2019-08-05

**Authors:** Margriet van Iersel, Rien de Vos, Marjon van Rijn, Corine H. M. Latour, Paul A. Kirschner, Wilma J. M. Scholte op Reimer

**Affiliations:** 1 ACHIEVE - Centre of Applied Research Faculty of Health, Amsterdam University of Applied Sciences, Tafelbergweg 51, 1105 BD Amsterdam, The Netherlands; 2 Department of Internal Medicine Section of Geriatric Medicine, Amsterdam UMC, Amsterdam, The Netherlands; 3 Centre of Evidence Based Education, Amsterdam UMC, Meibergdreef 9, 1105 AZ Amsterdam, The Netherlands; 4 Open University of the Netherlands, Valkenburgerweg 177, 6419 AT Heerlen, The Netherlands; 5 Department of Cardiology, Amsterdam UMC, Meibergdreef 9, 1105 AZ Amsterdam, The Netherlands

**Keywords:** Community care, Nurse education, Curriculum design, Perceptions, Career choice

## Abstract

**Background:**

The shift in healthcare to extramural leads to more patients with complex health problems receiving nursing care at home. However, the interest of baccalaureate nursing students for community nursing is moderate, which contributes to widespread labour-market shortages. This study investigates the effect of a more ‘community-care-oriented’ curriculum on nursing students’ perceptions of community care.

**Methods:**

A quasi-experimental quantitative survey study with a historic control group (*n* = 477; study cohorts graduating in 2015, 2016, and 2017; response rate 90%) and an intervention group (*n* = 170; graduating in 2018; response rate 93%) was performed in nursing students of a University of Applied Sciences in a large city in the Netherlands. The intervention group underwent a new curriculum containing extended elements of community care. The primary outcome was assessed with the Scale on Community Care Perceptions (SCOPE). The control and intervention group were compared on demographics, placement preferences and perceptions with a chi-square or T-test. Multiple regression was used to investigate the effect of the curriculum-redesign on nursing students’ perceptions of community care.

**Results:**

The comparison between the control and intervention group on students’ perceptions of community care shows no significant differences (mean 6.18 vs 6.21 [range 1–10], respectively), nor does the curriculum-redesign have a positive effect on students’ perceptions *F* (1,635) = .021, *p* = .884, R^2^ = < .001. The comparison on placement preferences also shows no significant differences and confirms the hospital’s popularity (72.7% vs 76.5%, respectively) while community care is less often preferred (9.2% vs 8.2%, respectively). The demographics ‘working in community care’ and ‘belonging to a church/religious group’ appear to be significant predictors of more positive perceptions of community care.

**Conclusions:**

Graduating students who experienced a more ‘community-care-oriented’ curriculum did not more often prefer community care placement, nor did their perceptions of community care change. Apparently, four years of education and placement experiences have only little impact and students’ perceptions are relatively static. It would be worth a try to conduct a large-scale approach in combination with a carefully thought out strategy, based on and tying in with the language and culture of younger people.

## Background

The shift from intramural to extramural healthcare in many Western countries is resulting in increasing numbers of patients receiving care in their own home environment [1]. These patients are, on average, growing increasingly older with more complex healthcare problems and chronic diseases [2]. This patient group is fast becoming an important population for healthcare professionals, resulting in an increase of care provided outside of a facility [3]. However, this development leads to the problem of labour market shortages, in part because many graduating students do not see the area of extramural or primary care as their preferred career choice [4–6]. The hospital with its acute and/or technically oriented care is most popular for students in both medicine [7–9] and nursing [10–13]. In both disciplines, a persistent long-term lack of interest in working with the elderly exists [14–16]. Working in primary care and/or community care also seems undervalued: students underestimate the qualifications and high academic standards required to ensure appropriate caregiving in this area [9, 16–18].

To meet the needs of society for highly educated healthcare professionals who aim to work in primary and/or extramural care, educators should challenge this imbalance and try to influence students’ career choices. Curriculum elements such as the content of lectures, courses, performing medical procedures, and contact with specific patient groups have an effect on the degree of interest and thus influence medical students’ career decisions [19]. This effect is also influenced by who the lecturer is [20]. A literature study by Pfarrwaller et al. [4] reveals that isolated modules and clerkships in the medical curriculum are not effective in stimulating career choice in primary care, leading to the recommendation to develop a longitudinal, multifaceted, primary care programme. In the nursing context, peers, senior students, and clinical experience appear to remain influential during study, while the influence of media and course content deteriorates further in the programme [13]. In both disciplines, working conditions as flexibility, income, and job security are also taken into consideration [21, 22].

Zooming in on community nursing, factors influencing a choice for this generalist home-based care in students are an older age, enjoyable patient relationships, care variety, and less perceived importance of workplace support and collaboration [5, 23]. Placements in the field are effective in stimulating students’ interest, as they strongly support their confidence and increase their competencies [24, 25]. Especially the more advanced students value the autonomy, the high level of knowledge necessary to handle the broad range of health problems, the holistic nature of the caregiving, and the opportunity to make their own decisions [24–27]. An isolated simulation in-school nursing programme in the UK, the ‘Community Challenge’, clarified the complexity of this field and was highly appreciated. It was not reported, however, whether it also effected students’ perceptions of the field [28].

In summary, the literature shows that exposure to aspects of caregiving in the community, both in school and in professional practice, potentially has a positive influence on students’ perceptions. Up to now, research mostly focussed on predicting those factors which influence students’ career choice and on students’ placement experiences. Longitudinal curriculum programmes are recommended in the medical context to increase the proportion of students choosing a primary care specialty. To date, however, there is no conclusive information available as to whether curriculum redesign as a whole can influence students’ perceptions in nursing and thus, can/will stimulate a choice for a future profession in community care.

### Aim of this study

The aim of this study is to investigate the effect of a longitudinal curriculum-redesign with extensive elements of community care on baccalaureate nursing students’ perceptions of community care.

## Methods

### Design

A quasi-experimental study with a historic control group and an intervention group was performed. The historic control group consisted of three student cohorts that underwent an older, more hospital-oriented nursing curriculum (study cohorts graduating in 2015, 2016, and 2017). The three cohorts were chosen because this was the maximum number of students that could participate after the newly developed instrument SCOPE (see section *Outcome and instrument*) had been validated and pilot-tested. The intervention group (one cohort graduating in 2018) underwent a redesigned curriculum with extensive elements of community care. The ‘Strengthening the Reporting of Observational Studies in Epidemiology’ (STROBE)-checklist for cohort studies [29], was used for analysis and reporting.

### A model for influencing perceptions

A psychological perspective was chosen as model for influencing students’ perceptions of community care, based on individual responses to specific stimuli (i.e., new curriculum in-school elements and experiences in professional practice during placements). The responses to these stimuli can be either *affective,* which are more or less transient positive and negative emotions that differ in intensity and valence, and/or *cognitive,* which are ideas about an object and, hence, how attractive the object (e.g., community care) is perceived to be [30]. This psychological perspective fits well with the DAGMAR-model - Defining Advertising Goals for Measured Advertising Results [31], a marketing communication model that focuses on the transaction and purchases performed by individuals. The DAGMAR model begins with awareness (e.g., that a career in community care exists), moves to comprehension (e.g., understanding what the career brings with it), then conviction (e.g., that the career somewhere is a good choice), and ends with action (e.g., making the career choice). It also highlights that, in this process, people progress through three stages: cognitive (thinking), then affective (feeling), and finally conative (doing). Therefore, influencing students’ perceptions means that a curriculum with more elements of community care will lead to increased knowledge of the field, leading to a notion of its possibilities and challenges, which will eventually lead to a growing appeal.

### Ethical considerations

The Ethical Review Board of the Open University of The Netherlands approved the study (reference U2014/07279/HVM). Students were informed via the digital learning environment of the institution as to a number of aspects of this study: the research projects’ purpose, information confidentiality and data access, and that non-participation would in no way impact their studies. This information was mentioned again during data collection in class, and verbal informed consent was obtained from all participants.

### Participants and data collection

Nursing students from a University of Applied Sciences in a large city in the Netherlands took part in the study. Data collection took place in students in the 4-year full-time BSc programme (240 EC, the normal duration of the Dutch BSc nursing programme). Students following specific educational pathways or programmes, and students that underwent only a part of the intervention due to enrolment in year two were excluded. Students in all four cohorts were approached for participation during allocated class time and, if not present, individually by email, in order to increase response. Data were collected for each cohort of students upon their graduation in May/June 2015, 2016 and 2017 (historic control group) and in 2018 (intervention group).

### Outcome and instrument

The outcome of the study was defined as ‘nursing students’ perceptions of community care’ measured by the Scale on Community Care Perceptions (SCOPE), a valid and reliable instrument (Cronbach’s α = .892), developed in the Netherlands [32, 33]. SCOPE contains, apart from items on demographics, thirty-three items in three subscales measuring the (1) affective component of community care perception, and two cognitive components, namely (2) perception of a placement in community care, and (3) perception of community nursing as a profession. Items in these scales range from 1 (negative adjective) to 10 (positive adjective). The option I don’t know is added in the placement and profession scale. The final two items of SCOPE measure the current placement preference in six healthcare areas (i.e., mental healthcare, elderly care, medical rehabilitation, care for mentally disabled, community care, and hospital care), and three aspects named in the earlier profession scale that primarily determine this preference. It might be noted that this questionnaire with its subscales measuring the affective and cognitive component of perceptions [34] ties in well with the approach of influencing perceptions based on the earlier described DAGMAR-model.

### The intervention: curriculum-redesign

The curriculum-redesign took the DAGMAR-model into consideration. It was intended to stimulate a positive interest in community care, and consisted of an integrative approach, based on: (1) the influence of lecturers, (2) placement experiences and (3) new educational elements in the in-school curriculum.

With regard to lecturers’ influence, the intervention focused on role modelling, lecturer expertise, and communication with students about healthcare areas. Concretely, community nurses were invited as guest-lecturers, presenting challenging patient cases from their daily practice. The teaching-team was expanded with new lecturers who were experts in community nursing either through experience and/or education. In a workshop to prepare curriculum redesign, reflecting on their own perceptions of healthcare areas, many lecturers noticed they implicitly or explicitly advocated their own professional history (often related to hospital care) as a reference point.

Second, efforts were made to ensure that placement in community care was considered a positive experience by the student, in the sense that it was seen to meet their learning needs. Mentors in the placement environment are crucial in influencing the nature and quality of placement experiences [17, 35, 36]. Finding good mentors in the workplace was hindered by labour market shortages of nurses with a suitable level of education, i.e., a bachelor’s degree. Therefore, management representatives from school and community-care organisations collaborated to ensure that students were mentored by the right professional role model.

Third, the in-school curriculum underwent a redesign. To understand what this redesign entails, it is important to have an idea of how the 4-year curriculum, by and large, is organised. The first two years are broad and generalist in nature and, thus, include theory about all types of patients in different contexts. In the second year, the students choose a minor programme for the third year, which is a one semester/20-week in-depth programme (30 EC) based on a specific theme, (e.g., science, global health), or a work field (e.g., mental health nursing). The other 20 weeks in year 3, as well as the first 30 weeks in year 4, contain two different placements, with the last and longest placement in year 4 in a preferred healthcare area. Generally speaking, the last two years allow students to give direction to their study, based on their own interests. These interests mostly relate directly to students’ preference for a field/ career choice.

**Fig. 1:**
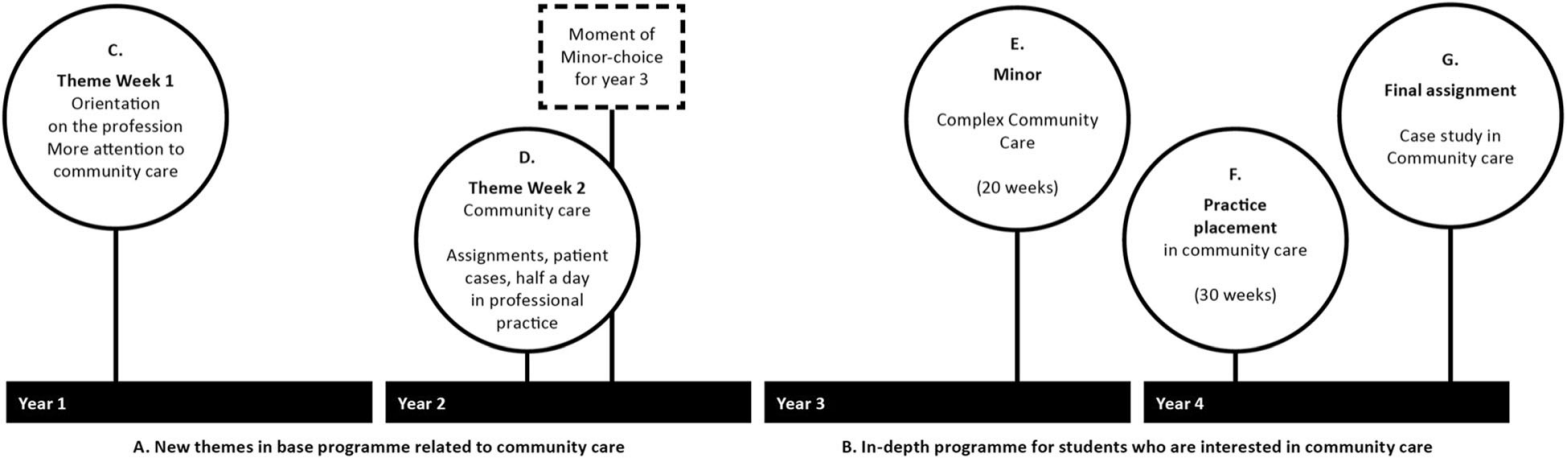
Curriculum-redesign stimulating a positive interest in community care

The first purpose of the curriculum redesign in the 4-year structure of this programme (see Fig. 1) was created to broaden students’ views on the nursing profession, showing that nursing is more than hospital care, and increase knowledge of community care. The course materials in this broad programme were scrutinised on how patient cases, used in the lessons, were presented. Although many of them did not refer to a specific context, more than 60 of the 110 cases were located in a hospital environment, compared to four patient cases receiving care in their own home. This aspect of the ‘hidden curriculum’, presenting the ‘hospital nurse in white’ as a common image, was corrected by adding more patient cases in the field of community care. Five new themes, derived from the new Dutch educational nursing profile with more elements of community care [37] were integrated in the broad theory programme in year 1 and 2, namely: (1) fostering patient self-management, (2) shared decision-making, (3) collaboration with the patients’ social system, (4) healthcare technology, and (5) allocation of care (see A in Fig. 1).

The second purpose of the curriculum-redesign was to offer an attractive and challenging in-depth programme in years 3 and 4 for students interested in community care, and thus pave the way to a choice for a final placement in community care, and possibly, a future career in this area [see B in Fig. 1]. The specific elements (circles C-G in the figure), related to the two periods A and B in the curriculum, are as follows:

C. Presentation of a broad picture of the nursing profession with special attention to community care in the introduction week at the start of the programme in year 1.
D. A ‘Community Care Week’ in the second year, with attractive assignments about patient cases, a digital game, speed dates with different types of community nurses (e.g., palliative care, nurse from a technical team, children home care). All students visit a nursing team in the community and, if possible, assist them in providing home care. This week is intentionally planned shortly before students’ minor-choice for year 3.
E. A new minor program in ‘Complex Community Care’ in year 3, including all aspects of community nursing on a higher level of complexity, such as population-based prevention, multimorbidity, interprofessional collaboration, professional leadership, and system-based communication.
F. A 30-week placement in year 4, facilitating the student in achieving all competencies required to fulfil the role of the independently working community nurse. In this period, students select a complex patient case from their professional practice for the final assignment.
G. A final assignment, designed as a case study, in which clinical reasoning plays a central role. The student analyses the chosen patients’ health problems and formulates a well substantiated advice for patient care, based on scientific evidence (evidence-based practice).

### Data analysis

The data on students’ perceptions of community care (SCOPE) were assessed on normal distribution, which showed that assumptions for using parametric statistics were fulfilled. Descriptive statistics were used to summarise student demographics. The control and experimental group were compared on demographics and perceptions of community care with a chi-square test or T-test. Students’ preferences for their final 30-week placement were compared with a chi-square test. Multiple regression was used to investigate the effect of the curriculum redesign on students’ perceptions. A calculation if the sample size was appropriate for this analysis was performed. Demographics that differed significantly between the two groups (step 2) and all other demographics in SCOPE, namely ‘sex’, ‘age’, ‘belonging to church or religious group’, ‘level of education’, ‘working/ has been working in community care (CC)‘, ‘family or friends working in CC ‘ and ‘receiving home care (or in family)’ (step 3), were added blockwise to the model. In the development of SCOPE, these factors were found to be potentially influential in students’ perceptions of community care [32, 33]. In all analyses, *P*-values smaller than .05 were considered as statistically significant. The data were analysed using IBM SPSS® version 25 (IBM Corporation, Armonk, NY).

## Results

### Response rate and comparison on demographics

Data from three respondents were excluded from the analysis as they only filled in a small part of the questionnaire. The historic control group from the three cohorts consisted of 477 students (response rate 90%), and the intervention cohort of 170 (response rate 93%). A comparison of demographics between these two groups shows statistically significant differences in one variable, namely ‘born outside the Netherlands’ (χ^2^ = 11.140, *p* = 0.001; Table 1).

**Table 1:** Comparison between historic control- and intervention groups on demographics

Student characteristics in % (*n*)	Historic control/Old curriculum (*n* = 477)	Intervention/New curriculum (*n* = 170)	Cases missing in total	Test-value	*P* (2-tailed)
Age in years (mean, SD)	23.1 (2.4)	23.0 (2.3)	0	T = .478	0.633
Sex (male)	11.3% (54)	11.2% (19)	0	χ^2^ = 0.030	0.959
Born outside the Netherlands	2.1% (10)	7.6% (13)	3	χ^2^ = 11.140	0.001*
Belonging to church/ religious group	14.6% (69)	20.7% (35)	4	χ^2^ = 3.479	0.062
Level of education			1	χ^2^ = 6.703	0.152
general secondary	68.8% (328)	67.0% (114)			
academic secondary	15.9% (76)	10.6% (18)			
vocational	13.6% (65)	20.6% (35)			
other	1.5% (7)	1.8% (3)			
Working/ has been working in CC	50.6% (241)	58.2% (99)	1	χ^2^ = 2.906	0.088
Family or friends working in CC	41.4% (197)	43.2% (73)	2	χ^2^ = 0.168	0.682
Receiving home care (or in family)	35.7% (169)	29.0% (49)	5	χ^2^ = 2.519	0.113

*P < 0.05; CC = community care

### Placement preferences

A comparison between the historic control group and intervention group on preferences for an area for the final placement, based on the selection of one of the aforementioned six healthcare areas, shows no statistically significant differences between the control and intervention group for any of the placement preferences. Community care placement was preferred by 9.2% vs 8.2% in the control and intervention group, respectively. The hospital remains the preferred area for a placement in both control (72.7%) and intervention group (76.5%) (Table 2).

**Table 2:** Comparison between historic control and intervention group on final placement preferences

Placement preferences in % (*n*)	Historic control/Old curriculum (*n* = 477)	Intervention/New curriculum (*n* = 170)	Cases missing in total	Test-value	*P* (2-tailed)
General hospital	72.7% (347)	76.5% (130)	0	χ^2^ = 0.897	0.343
Mental health care	12.8% (61)	8.8% (15)	0	χ^2^ = 1.900	0.168
Community care	9.2% (44)	8.2% (14)	0	χ^2^ = 0.150	0.698
Medical rehabilitation	3.6% (17)	3.5% (6)	0	χ^2^ = 0.000	0.983
Care for mentally disabled	1.0% (5)	2.9% (5)	0	NA*	NA*
Elderly care	0.8% (4)	0.6% (1)	0	NA*	NA*

*Not applicable: expected count < 5

### Perceptions of community care

A comparison between the historic control group and intervention group on perceptions of community care (SCOPE, total scale, subscales and items) shows a mean in the total scale of 6.18 vs 6.21 in the control and intervention group respectively, and the lowest score in the placement scale (mean 5.42 vs 5.51). The results show a very slight increase of the mean in all scales in the intervention group, but no statistically significant differences between the two groups for any of the scales, nor for each item.

However, the means of the separate items mutually differ substantially, showing that, in the historic control group as well as in the intervention group, the community care field is seen as ‘important’ (8.30 vs 8.58, respectively) and ‘meaningful’ (8.48 vs 8.60), but items representing a more personal attraction, such as ‘attractive’ (5.12 vs 4.86) and ‘fun’ (5.94 vs 5.73) score lower. The results in the placement scale show that students generally have moderate expectations (scores between 5.5 and 6.5), and even lower about contact with their mentor (4.67 vs 4.46) and time to evaluate with the mentor (5.15 vs 4.92). With regard to a profession in community care, students expect to care for many elderly patients in the field (8.75 vs 8.62) in a poor occupational work environment (4.33 vs 4.15), with little collaboration 4.74 vs 4.48), while carrying a lot of responsibility (8.48 vs 8.51). In addition to these negative perceptions of aspects of the caregiving, students see community nursing as a job with few advancement opportunities (5.27 vs 5.33) and a low status (4.96 vs 5.21) (Table 3).

**Table 3:** Comparison between historic control and intervention group on perceptions of community care (SCOPE: total scale, subscales, and per item)

Perceptions: range 1–10 in mean (SD) with mean values < 5.5 and > 8 in bold	Historic control/Old curriculum (*n* = 477)	Intervention/New curriculum (*n* = 170)	Cases missing^b^ (historic control + intervention)	Test-value t	*P* (2-tailed)
SCOPE: total scale (33 items)	6.18 (1.15)	6.21 (1.08)	0	- 0.338	0.735
Affective component scale (11 items)	6.53 (1.34)	6.55 (1.25)	0	- 0.117	0.907
Placement scale (5 items)	**5.42** (1.70)	5.51 (1.58)	14^a^	- 0.560	0.576
Profession scale (17 items)	6.54 (0.98)	6.57 (0.92)	0	- 0.303	0.762
Affective component scale					
Dull - interesting	6.01 (2.07)	5.81 (2.17)	1	1.072	0.284
Boring – fascinating	5.59 (2.00)	5.75 (2.11)	2	- 0.882	0.378
Unpleasant – pleasant	6.07 (1.92)	6.07 (1.98)	2	0.015	0.988
Annoying – agreeable	6.02 (1.85)	5.97 (1.80)	3	0.320	0.749
Uncomfortable – comfortable	5.81 (1.96)	5.65 (2.02)	7	0.916	0.360
Old fashioned – modern	6.53 (1.97)	6.87 (1.91)	3	- 1.928	0.054
Unimportant – important	**8.30** (1.95)	**8.58** (1.61)	2	- 1.883	0.061
Bad – good	**8.00** (1.84)	**8.14** (1.73)	2	- 0.814	0.416
Useless – meaningful	**8.48** (1.58)	**8.60** (1.65)	3	- 0.879	0.380
Unattractive – attractive	**5.12** (2.34)	**4.86** (2.29)	1	1.207	0.228
Stupid – fun	5.94 (2.15)	5.73 (2.27)	0	1.078	0.282
Placement scale^b^					
Very little – much variety in the caregiving	5.58 (2.38)	5.79 (2.42)	29	- 0.944	0.346
Very little – much contact with mentor	**4.67** (2.24)	**4.46** (2.04)	60	1.035	0.301
Very few – many opportunities to learn new things	5.68 (2.14)	5.96 (2.16)	24	- 1.430	0.153
My mentor will have very little – much time to evaluate	**5.15** (2.31)	**4.92** (2.18)	86	1.075	0.283
No – many possibilities to plan own learning activities	6.14 (2.29)	6.13 (2.11)	65	0.054	0.957
Profession scale^b^					
Very few – may enjoyable relationships with patients	7.72 (1.58)	7.77 (1.63)	15	- 0.378	0.706
Very little – much physically demanding work	7.24 (1.70)	7.20 (1.73)	8	0.258	0.796
Very little – much collaboration with colleagues	**4.74** (2.15)	**4.48** (2.03)	15	1.372	0.171
Very little – much collaboration with other disciplines	5.88 (2.26)	6.12 (2.16)	18	- 1.206	0.228
Very few – many technical skills needed	6.24 (2.08)	6.30 (1.85)	8	- 0.286	0.775
Very little – a lot of freedom of action	7.76 (1.66)	7.84 (1.73)	16	- 0.532	0.595
Very little – a lot of variety in the caregiving	5.79 (2.20)	5.85 (2.02)	13	- 0.321	0.748
A poor – good occupational work environment	**4.33** (1.94)	**4.15** (1.96)	46	1.043	0.297
Very little – plenty of individual responsibility	**8.48** (1.25)	**8.51** (1.20)	6	- 0.308	0.758
No – continual feelings of work pressure	7.34 (1.75)	7.40 (1.53)	18	- 0.407	0.684
Very few – plenty of complex patient care needs	6.09 (2.05)	6.02 (1.84)	19	0.361	0.718
Very few – only elderly patients	**8.75** (1.23)	**8.62** (1.23)	3	1.252	0.211
Low – high status work	**4.96** (1.88)	**5.21** (1.81)	34	- 1.435	0.152
No – a lot of possible health improvement for the patient	6.37 (1.91)	6.49 (1.79)	29	- 0.737	0.461
Very few – many enthusiastic colleagues	6.30 (1.92)	6.29 (1.71)	61	0.070	0.944
Very few – much contact with family/ kin	7.70 (1.79)	7.90 (1.62)	14	- 1.213	0.226
No – many opportunities for advancement	**5.27** (2.14)	**5.33** (2.23)	41	- 0.271	0.787

^a^ Cases with no data in the placement scale or with the option ‘I don’t know’ in all 5 items

^b^ The option ‘I don’t know’ (value 11) in the placement and profession scale is excluded in the calculation of the mean and defined as missing, which explains the larger/ fluctuating numbers of missing values in the placement and profession scale

### Effect of curriculum-redesign on nursing students’ perceptions of community care

To measure the effect of curriculum redesign on nursing students’ perceptions of community care (SCOPE), controlling for difference on demographics, a multiple linear regression was carried out. The average variance inflation factor (VIF) was very close to 1, showing that the assumption of no multicollinearity was true for the model [38]. The required sample size for the regression analysis was calculated, based on a power of .80 and an alpha of .05, with the rule of thumb ‘required *N* ≥ 50 + 8m (with m being the number of predictors)’ [39], indicating that the sample of *N* = 647 is more than adequate. The main model/ step 1, predicting students’ perceptions of community care (SCOPE) from the type of curriculum (historic/intervention), shows no statistically significant difference in perception, *F* (1,635) = .021, *p* = .884, and a low explained variance R^2^ = < .001. In the second step, the variable ‘born outside the Netherlands’ (being statistically different in the control and intervention group, see Table 1) was added to the model. This model did not significantly differ from the main model *F* (2,634) = .124, *p* = .883, R^2^ = < .001, indicating that the variable ‘born outside the Netherlands’ did not have an influence on students’ perceptions of community care. In step 3, a model in which all potentially influential variables (see Table 1) were added, significantly explained the perceptions *F* (11,625) = 6.195, p = < .001, R^2^ = .098. In this model, although the explained variance of .098 remains relatively low, not the curriculum, but the variables ‘belonging to a church/ religious group’ (*p* = .027) and ‘working/ has been working in community care’ (*p* = < .001) were significant predictors of students’ perceptions of community care (Table 4).

**Table 4:** Multiple regression analysis for the effect of curriculum on nursing students’ perceptions of community care

	*B*	*SE B*	β	95% Confidence Interval
**Step 1**				
Constant	6.190	.052		6.089–6.291
Curriculum	.015	.101	.006	−.183–.213
**Step 2**				
Constant	6.188	.052		6.086–6.289
Curriculum	.009	.101	.004	−.190–.209
Born outside the Netherlands	.119	.250	.019	−.372–.610
**Step 3** ^a^				
Constant	5.238	.487		4.282–6.194
Curriculum	−.047	.098	−.019	−.240–.146
Born outside the Netherlands	.000	.248	.000	−.487–.486
Level of education				
secondary	Ref	Ref	Ref	Ref
higher secondary	−.098	.126	−.031	−.344–.149
professional	.227	.133	.074	−.033–.487
other	.025	.376	.003	−.713–.763
Sex (male)	−.116	.140	−.033	−.391–.159
Age	.023	.022	.050	−.019–.066
Belonging to church/ religious group	.263	.119	.086*	.030–.496
Working/ has been working in CC	.558	.087	.249*	.387–.728
Family or friends working in CC	.128	.087	.056	−.044–.300
Receiving home care (or in family)	.087	.092	.037	−.094–.268

*Note. R*^*2*^ = < .001 for Step 1; Δ *R*^*2*^ = < .001 for Step 2 (*p* < .05); Δ*R*^*2*^ = .098 for Step 3 (*p* < .05)

*p < .05

^a^In the development of SCOPE, these factors were found to be potentially influential in students’ perceptions of community care [32, 33]

## Discussion

The objective of this study is to investigate the effect of a curriculum with more elements of community care on nursing students’ perceptions of community care. The association between this redesign and nursing students’ preferences for a final placement, which is often a predictor for the students’ career choice, were also studied. The results show that students’ placement preferences in the group that went through the new curriculum hardly differ from the control group, nor does the curriculum-redesign significantly effect students’ perceptions. With regard to influential student characteristics, differences in students’ perceptions of community care could possibly be explained by the variables ‘working/has been working in community care’ and ‘belonging to a church or religious group’.

This study reveals that, in this institution, the curriculum-redesign was not effective in creating more interest for the field of community care, despite the fact that the intervention was designed as a holistic approach and longitudinal programme; the only way to potentially stimulate positive interests for less popular healthcare areas [4]. Since the intervention is complex and takes place over a longer period, it is likely that different kinds of influences and explanations play a role. First, specific interventions may be influential only for a short period, which means that the effect fades out. Stand-alone courses and other interventions promoting lesser popular healthcare areas have proved to be successful in stimulating students’ interest [28, 40] but it is unclear if there is a long-term effect as well. A study by Lewis et al. (2018) revealed that students would like to have more content on community nursing in the curriculum, but only *after* they experienced an interesting placement in the field [41], which supports the importance of positive placement experiences. Second, despite the new themes on community care in the generalist part in the curriculum in year 1 and 2, other pathways for hospital care in year 3 and 4 in the curriculum, and the start of a new master in Critical Care in this university may function as a competitive offer. Third, influences in the ‘hidden curriculum’ may have more impact on students, such as disappointing experiences in the practice of community nursing, leading to ‘badmouthing’ between peers, an aspect significantly influencing student’s career choices [8]. Fourth, workforce (and workload) problems in the community care field mentioned in the media, and the long-term stereotypical notion of the ‘nurse in white’ working in the hospital [6, 7, 42] are proved to be constraining factors contributing to the lack of growing interest. These are aspects of societal and cultural influences that may be of greater influence.

The variable working/ has been working in community care appears to positively influence students’ perceptions of the field. In the overall number of 647 participating students, about half provides or did provide care in the community in different situations and roles; some of them in the role of a nurse with a diploma in secondary nursing before study, others during study in a placement or student job. In general, experience in caregiving predicts the choice for a nursing career [43] and there is an established body of knowledge about the impact of placement experiences on career choice [12, 13, 16, 19, 44]. If these experiences are acquired in community nursing, they are helpful in clarifying the role and content of this practice, and thus counterbalance widespread misconceptions [17, 35, 41]. The slower pace of this work environment, often mentioned as valuable since the mentoring nurse can spend additional time with a student [36], also helps to clarify issues related to the profession [17, 27]. All these aspects may influence students’ perceptions positively, even if the caregiving is performed on a lower level of complexity than that of the bachelor-educated nurse, an aspect that potentially could have a negative influence. Apparently, student’s experiences in community nursing gives them a sense of the attractiveness of the various aspects of caregiving ‘behind people’s front doors.’

With regard to the positive influence of the variable ‘belonging to a church and/or religious group’ on students’ perceptions, research on the relationship between religiosity and career choice in nursing students is lacking. Although religious nursing students appear to be more idealistic [45] and altruism and caring are the main motives for choosing a nursing career [46, 47], no relation between altruism and religiosity has been found [48], nor has any relation between religiosity and working in the community been established. Clearly however, this is not an area of influence for educators with regard to students’ career choices.

Taking all this into consideration, it seems that influencing perceptions of community care with a curriculum, despite the comprehensive approach undertaken, is not an easy ambition. This is confirmed by the fact that students’ perceptions, here measured at the end of their programme, are generally in line with the results of a large survey on Dutch first-year students’ perceptions of community care, in positive as well as in negative aspects [5]. It appears that four years of education and placement experiences had only little impact and that students’ perceptions are relatively static. It would be worth a try to conduct a large-scale approach in combination with a carefully thought out strategy, which is based on and ties in with the language and culture of younger people. If the societal image of the role of the nurse, increasingly working in extramural care, can be modified, it is likely that the perceptions of young people choosing for an education in nursing will follow, which will hopefully lead to different career choices.

### Strengths and limitations

One strength of this study is its relatively large sample size and high response rate compared to many other studies. Another strength is that the intervention was designed as a holistic/longitudinal programme. Although the choice for this integrated programme is not called into question, a limitation is that it is difficult to control, specifically in a study design where a distinction between a control- and intervention group is required. The way education is organised in student cohorts progressing separately through their programme makes it relatively simple to provide a new cohort with other theoretical content. However, with regard to the influence of lecturers and mentors, it is likely that different messages to students about the opportunities of community care is closely interlinked with a process of growing awareness in themselves. Although this awareness usually evolves in the right direction, it is difficult to establish if this communication, as part of the intervention, was not limited exclusively to the intervention group, and thus possibly was received, to a greater or lesser extent, by the control group as well. Finally, the results of this study have limited generalizability as the study was conducted at a single institution.

### Implications for further research

This study-design focusses on the effect of a curriculum as a whole, which leaves questions about the effect of separate parts of this design unanswered. To measure how students’ perceptions develop while progressing through the 4-year redesigned programme, and see if and how separate elements have their influence, a longitudinal approach is necessary. Therefore, this will be carried out in a subsequent study. Insights about if and how specific curriculum elements influence students’ perceptions positively will be helpful in formulating recommendations for further steps in curriculum-design.

A second recommendation, as research on this topic is lacking, is to study what ‘dosage’ in interventions in the generalist/ broad part of the curriculum is suitable for stimulating student’s interest for a specific healthcare area. It is likely that too much attention on one area leads to an opposite effect when students feel somewhat pushed or even manipulated. As educators have a role in supporting students to make well-informed career choices instead of putting pressure on them, this is a practical (it will not be effective) and an ethical point of interest.

## Conclusion

The numbers of graduating nursing students entering the field of community care is limited, which is an important reason for educators to stimulate student’s interest for the field. This study offers insights into choices made in designing a longitudinal nursing curriculum with more elements of community care. However, graduating students who had gone through this new curriculum did not have other placement preferences, nor did their perceptions of the community care field change significantly. More research is needed to examine how separate interventions in the curriculum work. A well-considered large-scale strategy that ties in with the language and culture of younger people with the purpose to promote positive perceptions of community care could be a useful addition.

## Data Availability

The dataset supporting the conclusions of this article is available in the Figshare repository: 10.21943/auas.7700840.v1

## Abbreviations

BSc: Bachelor of Science
CC: Community Care
DAGMAR: Defining Advertising Goals for Measured Advertising Results
EC: European Credits
SCOPE: Scale on Community Care Perceptions
STROBE: Strengthening the Reporting of Observational Studies in Epidemiology
